# New developmental evidence supports a homeotic frameshift of digit identity in the evolution of the bird wing

**DOI:** 10.1186/1742-9994-11-33

**Published:** 2014-04-12

**Authors:** Miguel Salinas-Saavedra, Cristian Gonzalez-Cabrera, Luis Ossa-Fuentes, Joao F Botelho, Macarena Ruiz-Flores, Alexander O Vargas

**Affiliations:** 1 Laboratorio de Ontogenia y Filogenia. Departamento de Biología, Facultad de Ciencias, Universidad de Chile, Las Palmeras 3425, Ñuñoa, Santiago, Chile

## Abstract

**Background:**

The homology of the digits in the bird wing is a high-profile controversy in developmental and evolutionary biology. The embryonic position of the digits cartilages with respect to the primary axis (ulnare and ulna) corresponds to 2, 3, 4, but comparative-evolutionary morphology supports 1, 2, 3. A homeotic frameshift of digit identity in evolution could explain how cells in embryonic positions 2, 3, 4 began developing morphologies 1, 2, 3. Another alternative is that no re-patterning of cell fates occurred, and the primary axis shifted its position by some other mechanism. In the wing, only the anterior digit lacks expression of *HoxD10* and *HoxD12*, resembling digit 1 of other limbs, as predicted by 1, 2, 3. However, upon loss of digit 1 in evolution, the most anterior digit 2 could have lost their expression, deceitfully resembling a digit 1. To test this notion, we observed *HoxD10* and *HoxD12* in a limb where digit 2 is the most anterior digit: The rabbit foot. We also explored whether early inhibition of *Shh* signalling in the embryonic wing bud induces an experimental homeotic frameshift, or an experimental axis shift. We tested these hypotheses using DiI injections to study the fate of cells in these experimental wings.

**Results:**

We found strong transcription of *HoxD10* and *HoxD12* was present in the most anterior digit 2 of the rabbit foot. Thus, we found no evidence to question the use of HoxD expression as support for 1, 2, 3. When Shh signalling in early wing buds is inhibited, our fate maps demonstrate that an experimental homeotic frameshift is induced.

**Conclusion:**

Along with comparative morphology, HoxD expression provides strong support for 1, 2, 3 identity of wing digits. As an explanation for the offset 2, 3, 4 embryological position, the homeotic frameshift hypothesis is consistent with known mechanisms of limb development, and further proven to be experimentally possible. In contrast, the underlying mechanisms and experimental plausibility of an axis shift remain unclear.

## Background

**Figure 1:**
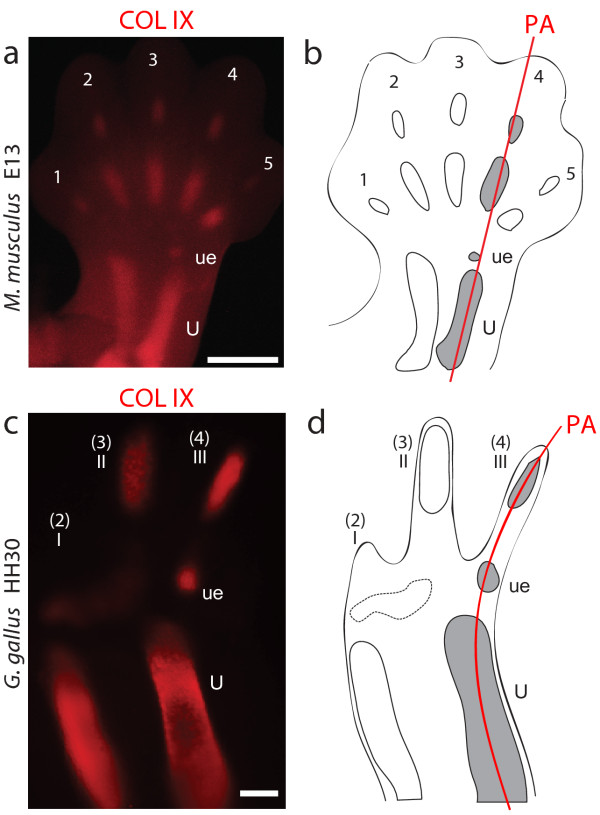
**The embryological position of the wing digits is 2, 3, 4.** Collagen type 9 whole mount immunofluorescence showing relative positions of embryonic skeletal elements in a non-controversial limb, and in the bird wing. **(a, b)** In a pentadactyl limb, like that of the mouse (*Mus musculus*), the cartilage of digit 4 is spatially aligned with the ulnare and the ulna, conforming the primary axis (red line). **(c, d)** In the embryonic wing of the chicken (*Gallus gallus*), the cartilage of the posterior digit occupies the same position (red line), suggesting it is a digit 4, as in 2, 3, 4. Scale bars: **a**, **c**—500 μm.

**Figure 2:**
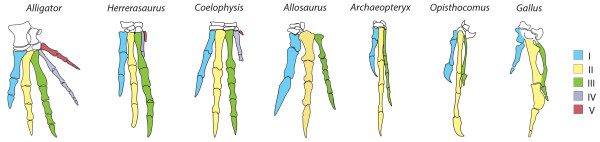
**Morphological data supports 1, 2, 3 in the bird wing.** The evolution of digit morphology is well documented and shows the reduction and loss of digits 4 and 5 in taxa sharing a successively more recent common ancestor with modern birds (from left to right). The digits on the tridactyl wing of mesozoic birds like *Archaeopteryx* still had the same number of phalanges than digits 1, 2, 3 of dinosaurs and other reptiles.

The identification of the three digits of the avian wing can be described as a scientific “crisis” because of conflicting signals from two reliable, often-used data sources on homology. In the embryonic wing, the position of the early digit cartilages suggest 2, 3, 4: The posterior digit is the first digit formed, in spatial alignment with the ulna and ulnare, conforming the “primary axis” that develops into digit 4 in non-controversial limbs [1-3] (Figure 1). Within palaeontology, however, wing digits are traditionally labelled 1, 2, 3 based on several morphological resemblances to these digits in other reptiles, such as the number of phalanges [4]. Fossils documenting the dinosaur-bird transition show how posterior digits 4 and 5 became reduced and subsequently lost in evolution [5-7] (Figure 2). Some authors have suggested that the digits of early tetanuran dinosaurs (for instance, *Allosaurus* in Figure 2), which are ancestors of birds, could actually be 2, 3, 4 [8-10]. However, since 1, 2, 3 gains more support from morphological evidence [10-13], the hypothesis that tetanuran digits are 2, 3, 4 relies heavily on the assumption that development in birds (living tetanurans) univocally supports 2, 3, 4 [10]. In fact, developmental evidence to support 1, 2, 3 is also available. In non-controversial limbs, the embryonic expression of *HoxD10*, *HoxD11* and *HoxD12* is absent only in digit 1. Likewise, in the wing, these genes are not expressed in the most anterior digit, as in 1, 2, 3 [14-16]. However, it is argued that this evidence could be equivocal [10,17]. Lack of expression of *HoxD10*, *HoxD11*, and *HoxD12* could relate to the position of whichever is the most anterior digit: Thus, if a limb loses digit 1 in evolution, digit 2 could cease to express these genes, creating the wrong impression it is a digit 1. This argument has been named the MAD (Most Anterior Digit) hypothesis [17,18]. To address this concern, we have observed transcription of HoxD genes in a limb where digit 1 is unequivocally absent, such that digit 2 is the most anterior digit: The rabbit foot.

The early expression of Shh in the wing bud is also important to the debate on digit identity [19]. A spatio-temporal gradient of posteriorly expressed *Shh* protein patterns the antero-posterior axis of the limb bud, with greater concentrations and longer exposures determining more posterior digit identities [20-22]. In the mouse, endogenously expressed *Shh* is absent from the precursors of digits 1, 2, and the anterior half of digit 3 [23] which is also the case for the anterior, middle and posterior digits of the wing, respectively, providing support for 1, 2, 3 [19,24]. Assuming the evidence for 1, 2, 3 identity is correct, different hypotheses could explain the 2, 3, 4 embryonic position. A decrease in the postero-anterior gradient of Shh signal, either by reduced concentration and/or reduced exposure time, could have induced a homeotic frameshift in evolution, such that cartilages in positions that previously became 2, 3, 4 began developing the adult morphologies of digits 1, 2,3 [5,19,25]. Alternatively, a shift in the position of the primary axis occurred, without any re-patterning of cell fates (the “axis shift” hypothesis [24,26]). Experimental inhibition of early *Shh* signaling leads to bidactyl wings, in which the posterior digit is missing [27,28]. In these bidactyl wings, the middle digit develops in line with the primary axis [25], but this experiment has been interpreted differently, in favour of the homeotic frameshift hypothesis [25] or the axis shift hypothesis [24]. To clarify this controversy, we have marked cells and fate-mapped them, in both control wings and wings under *Shh* inhibition.

## Results

### HoxD expression in a limb that has lost digit I does not resemble the wing

**Figure 3:**
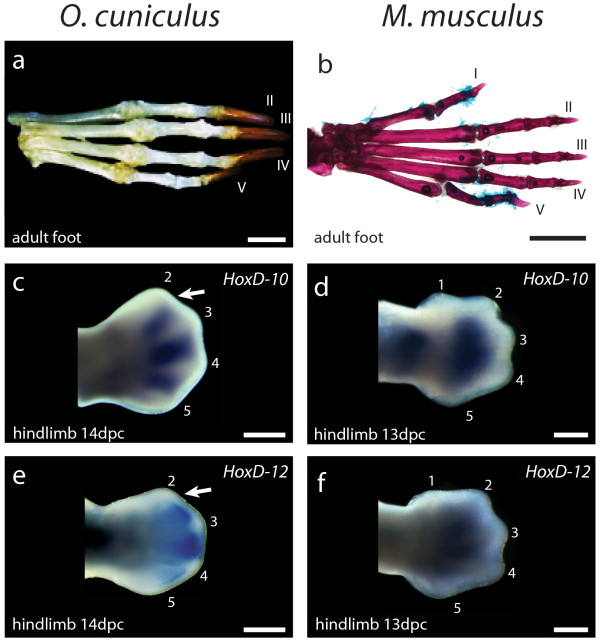
**HoxD expression of digit 2 in a limb that has lost digit 1 remains distinct from digit 1.** It is argued that upon loss of digit 1 in evolution, the new most anterior digit, digit 2, may cease to express *HoxD10* and *HoxD12*, thus resembling digit 1. **(a, b)** Adult morphology of the foot of the rabbit *Oryctolagus cuniculus* compared to the mouse *Mus musculus*. In the tetradactyl foot of the rabbit*,* the long metatarsals and triphalangeal morphology of the digits are typical of digits II-V of mammals. The only missing digit is the biphalangeal digit 1, which in other Glires (such as mouse) has only two phalanges, and a much shorter metatarsal. **(c-f)** Expression in the embryonic foot of the rabbit and mouse. **(c, e)***HoxD10* and *HoxD12* expression is present in the posterior interdigital mesenchyme (PIDM) of digit 2 of the rabbit foot. **(d, f)** Expression is absent from the PIDM of digit 1 of the mouse foot, making it clearly distinct from digit II of the rabbit. Scale bars: **a**, 5—mm; **b**—5 mm; **c**, **e**—500 μm; **d**, **f**—500 μm.

Digit identity is determined at late stages, when cartilaginous digital rays and their interdigital mesenchyme are clearly recognizable [29,30]. The interdigital mesenchyme immediately posterior to a digital ray (PIDM, Posterior Inter Digital Mesenchyme) is a signalling center that is crucial to the determination of the morphological identity of a digit [29,30]. In limbs where digit identity is non-controversial, *HoxD10*, *HoxD11* and *HoxD12* may or may not be strongly transcribed in the anterior aspect of digit 2, but are always strongly transcribed in its PIDM [15,16]. In the adult foot of the rabbit (*Oryctolagus cuniculus*) a triphalangeal digit 2 is the undisputed most anterior digit (Figure 3a). Only four digital rays are formed in the embryo. The absence of the digital ray and PIDM of digit 1 is consistent with observations that digit loss in mammals does not proceed by secondary developmental reduction of digital rays after their formation. Rather, digits are reduced through evolutionary modifications in the early developmental patterning of limbs [31]. Digital rays of missing digits fail to form along with those of other digits: Only a small metacarpal vestige may appear at a later stage [31-33]. We found that at 14 days post coitus (14 dpc) *HoxD10* and *HoxD12* are strongly transcribed in the PIDM of digit 2, despite the fact that in this species, this is the most anterior digital ray formed (Figure 3c, e). This does not resemble whole mount in situs of the anterior digit of the wing, where transcripts are undetectable in its posterior interdigital mesenchyme. In the pentadactyl foot of the mouse (*Mus musculus*) (Figure 3b), these genes are strongly transcribed in the PIDM of digit 2, and undetectable in the PIDM of digit 1, as expected (Figure 3d, f).

### Inhibition of *Shh* signalling produces an experimental homeotic frameshift in the wing

**Figure 4:**
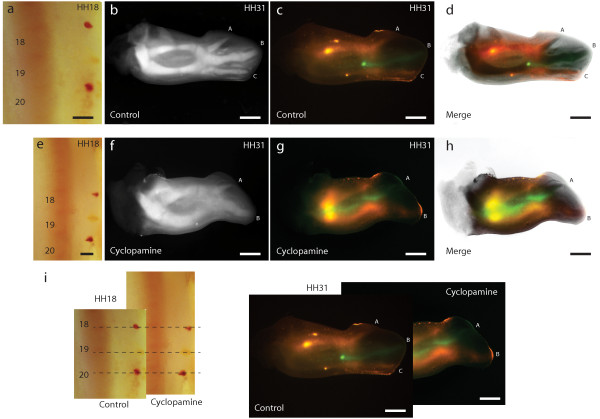
**Inhibition of *Shh *signalling produces an experimental homeotic frameshift. (a)** Triple injection with DiI (Red) and DiO (Green) along the antero-posterior axis at stage HH18, and subsequent fates at HH stage 31 **(b, c, d)**. **(e)** Triple injection with DiI and DiO at stage HH18, and their fate at HH31 after cyclopamine application **(f, g, h)**. Inhibition of *Shh* signaling results in bidactyl wings where a homeotic frameshift has occurred. Cells in positions that would normally give rise to middle **(B)** and posterior **(C)** digit morphologies now give rise to anterior **(A)** and middle **(B)** digit morphologies. Cells that would normally give rise to the anterior digit fail to develop into a digit. The posterior digit morphology is absent. **(d)** Merge of **b** and **c**. **(h)** Merge of **f** and **g**. **(i)** Comparison of injections made in control and cyclopamine-treated embryos. Scale bars: **a**, **e**—300 μm; **b**, **c**, **d**—1 mm; **f**, **g**, **h**—1 mm.

**Figure 5:**
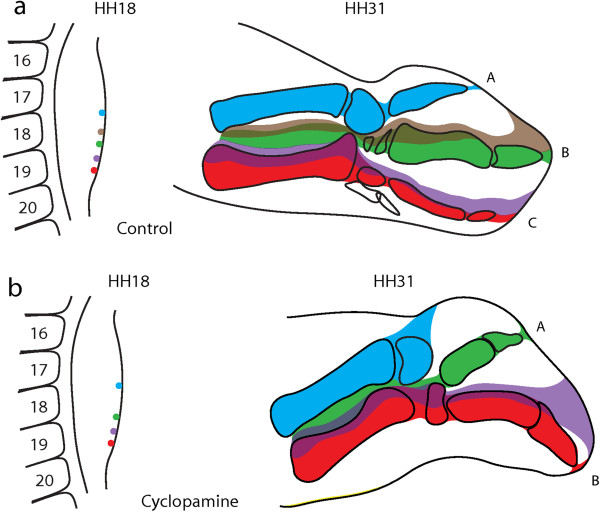
**Results of single injections at HH18 and their fate at control and cyclopamine-treated HH31 wings.** Asides from the triple injection shown in Figure 4, we performed numerous single injections that confirm an experimental homeotic frameshift is induced by inhibition of Shh signalling. **(a)** In control embryos, labelled cells near the limit between somites 17/18 developed into digit A (blue, n = 11). Labelled cells near the limit between somites 18/19 were allocated to digit B (green and brown, n = 16). Labelled cells near the limit between somites 19/20 formed digit C (red and purple, n = 30). **(b)** In cyclopamine-treated embryos, labelled cells near the limit between somites 17/18 allocated anterior to the cartilage of any digit (blue, n = 5). Labelled cells near the limit between somites 18/19 were allocated to a digit A (green, n = 10). Labelled cells between somites 19/20 develops into digit B (red and purple, n = 26).

**Figure 6:**
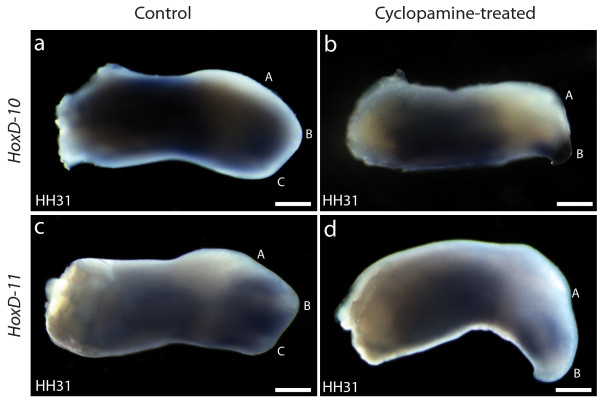
**Expression of *HoxD10 *and *HoxD11 *is posteriorly shifted in cyclopamine- treated HH31 wings. (a, b)***HoxD10* and **(c, d)***HoxD11* expression continue to be absent in the development of a digit 1 morphology, despite the fact it develops from cells in a position that in control wings expresses these genes and develops a digit 2 morphology. The simultaneous shift of HoxD expression and morphology is also implied in the hypothesis of an evolutionary homeotic frameshift. Scale bars: **a**, **d**—1 mm.

For convenience, in this section we will refer to the anterior, middle and posterior digit morphologies of the wing digits as A, B, and C, respectively. Previous work has shown that down-regulation of *Shh* signaling by applying cyclopamine at stage 19 (presumably including late stage 18- early stage 20) results in a bidactyl wing where only digits A and B are formed [27,28]. In these bidactyl wings, digit B develops in line with the primary axis [25]. We studied cell fate by placing DiI (Red) and DiO (green) injections at different positions along the antero-posterior axis of stage HH18 wing buds, immediately before the application of cyclopamine. Figure 4a shows a triple injection of DiI, DiO, and DiI again, in a control wing, at positions that are later observed to allocate to digits A, B, and C, respectively, of the same wing at HH 31 (Figure 4b, c, d). This result is in agreement with previously published fate map studies of the chicken wing [24,34]. Figure 4e shows the result of a triple injection at equivalent positions, immediately previous to cyclopamine application. At stage HH31 the anterior position (red) is later observed to allocate to anterior tissue that does not give rise to any digit, while the middle (green) and posterior (red) positions now give rise to digits A and B, respectively (Figure 4f, g, h). This result is consistent with numerous single injections (technically easier to perform than triple injections). The results of single injections are summarized in Figure 5. DiI injections at the boundary between somites 19 and 20, which normally develop into digit C (Figure 5a, indicated by red and purple, n = 30) consistently give rise to digit B in cyclopamine-treated wings (Figure 5b, indicated by red and purple, n = 26). Injections at the level of the boundary between somites 18 and 19, which normally give rise to digit B (Figure 5a, indicated by green and brown), give rise to digit A (Figure 5b, indicated by green, n = 10), and cells at the boundary between somites 17 and 18, that in control wings become digit A (Figure 5a indicated by blue) are found in anterior cells that fail to develop into a digit (Figure 5b, indicated by blue, n = 5). We also confirmed that, as reported previously for *HoxD12*[26], the expression of *HoxD10* and *HoxD11* in bidactyl wings is absent in the PIDM of digit A (Figure 6), despite the fact it now develops one digital position closer to the primary axis. Our results conclusively demonstrate the re-specification of cells in cyclopamine-treated wings. Alternatives such as cell re-allocation, the death of the precursor cells of digit C, or the fusion of digits B and C [35] are effectively discarded. Previously, it was shown that in the experimental bidactyl wings, the primary axis develops into a digit B morphology [25]. Our new results confirm the primary axis in bidactyl wings adequately reflects the early position of digit precursor cells before cyclopamine application. Loss of digit C is not the result of cell death: Rather, digit C morphology fails to develop at the position of the primary axis, presumably due to insufficient *Shh* signal, which leads to a digit B morphology instead. Previous work has also fate-mapped cells in cyclopamine-treated wing buds. One of these studies delivered results apparently different from ours, suggesting that in bidactyl wings, the posterior digit is a “fused composite” of cells that become B and C in control wings [35]. However, fate was observed too early, before digit identity and the experimental phenotype could be recognized with certainty. Importantly, cyclopamine was applied at stage 20, rather than stage 19, with increased chances of producing non-bidactyl wings where digits B + C are fused [26]. Another study that applied cyclopamine at stages 19–20, and observed fate in well-differentiated digits [24], provided results consistent with ours. However, this study focused only on the fate of the most posterior (ZPA) cells and favoured an “axis shift” interpretation. The new, fully informative data set presented in our study, including triple injections, is required to properly recognize the occurrence of an experimental frameshift.

## Discussion

If wing digit identity were 2, 3, 4, coinciding directly with embryological position, both the homeotic frameshift and axis shift hypotheses would be unnecessary. This could be the case if support for 1, 2, 3, were equivocal. Our data from the rabbit foot produced no evidence to question the use of HoxD expression as support for 1, 2, 3. The MAD (Most Anterior Digit) hypothesis claimed that upon loss of digit 1, a most anterior digit 2 would take over the HoxD signature expression of digit 1, as a result of intrinsic properties of Shh signalling and HoxD regulation [17,18]. If this were indeed such an inescapable outcome of limb development, HoxD expression in the wing would be trivially as expected for 2, 3, 4. However, the rabbit foot suffices to demonstrate that the MAD hypothesis does not always apply. At best, the MAD hypothesis is still possible, but unsupported by any actual empirical case. Our result is further relevant considering that HoxD regulation is largely understood from studies made in the mouse, a fairly close relative of the rabbit. It could be argued that the rabbit is too distantly related to birds and thus irrelevant to discuss the plausibility of events in the evolution of that lineage. However, developmental biologists constantly integrate information from mouse and chicken, even though they are distant relatives. This practice is informative because molecular mechanisms of limb development (Shh expression, HoxD cluster structure) are highly conserved. The closest living relatives of birds are the Crocodylia, but they cannot test the MAD hypothesis since none presents reduction of digit 1. Digit 1 reduction is found among squamates, but these have accumulated unusually large numbers of transposable elements in their Hox clusters [36]. In fact, the mammalian HoxD cluster is more comparable to that of birds. For now, the rabbit foot provides the only available data for HoxD expression in limbs where loss of digit 1 is non-controversial. Even if a small field with no *HoxD10-12* is still present in the rabbit foot, it is clearly larger in the bird wing, engulfing the PIDM of the anterior digital ray. Thus, comparison to the rabbit foot provides no support for the occurrence of digit 1 reduction in the wing.

Any trait that is proposed to mark the identity of a given digit is strengthened when the association is maintained across different limbs: The greater the sample, the better, demonstrating great evolutionary conservation. Lack of *HoxD10*, *HoxD11* and *HoxD12* in digit 1 has been confirmed in the chicken foot and both the hand and foot of the mouse [14]. Lack of *HoxD11* in digit 1 has also been confirmed in the hand and foot of the alligator and of the three-toed skink [15,37]. The new data from the rabbit foot further supports the use of HoxD expression to identify digit 1. As with the HoxD genes, an extended sample of limbs, including limbs that lost digit 1, will test the validity of other genes suggested as markers of digit 1 identity, such as *Zic2* and *Lhx9*. These are expressed in the anterior digit of the wing and digit 1 of the chicken foot [16]. Further limb sampling could also strengthen the case that digits 1,2, and most of digit 3 are derived from cells that do not express *Shh*, as in both the hand and foot of mouse and chicken [19,23,24]. For now, HoxD expression remains the best-documented line of developmental evidence to support 1, 2, 3.

Developmental evidence for 1, 2, 3 should not be simply dismissed. A recent quantitative study presented parsimony analysis, allegedly supporting 2, 3, 4 identity in the lineage leading to birds ever since early tetanuran dinosaurs like *Allosaurus*[10]. However, developmental evidence was only used to assume 2, 3, 4, “a priori” in *Archaeopteryx*, (the only Avialae included in that analysis) and thus code all morphological traits as if present on digits 2, 3, 4 of this taxon. This assumption managed to reverse the result of parsimony analysis of morphological data, which otherwise supports the traditional 1, 2, 3 identification of tetanuran digits [10]. Current molecular-developmental evidence for 1, 2, 3 questions this and any other analysis constructed on the assumption that development univocally supports 2, 3, 4, which only reflects information regarding embryological position.

The evidence for 1, 2, 3 strengthens the case that the position of the primary axis is not related to digit identity by any direct mechanism of causation [38,39]. The argument has been made that the primary axis is non-significant to the extent that digits are simply 1, 2, 3, and no homeotic frameshift hypothesis is necessary [24]. We think this view is extreme: While the primary axis is not directly related to digit identity, it remains a reliable indicator of relative position among the cartilaginous elements of the embryonic limb. Additionally, our fate maps confirm that in control and cyclopamine-treated wings, the position of a digit with regard to the primary axis directly reflects the earlier antero-posterior position of its precursors at autopod patterning stages. In non-controversial limbs, the digit cartilage at the primary axis consistently gives rise to digit 4, rather than digit 3. Thus, an explanation is still required on how morphological identity and gene expression in the wing have shifted their position towards posterior. Accumulated knowledge on the molecular-developmental mechanisms of digit patterning through a spatio-temporal posterior gradient of *Shh* signaling has provided a framework in which a homeotic frameshift is readily conceivable [5,25]. It is further significant that reduced *Shh* signaling using cyclopamine in fact produces such an experimental frameshift. This discards characterizations of the frameshift as an “awkward” or “ad hoc” auxiliary hypothesis, with no reason of being beyond explaining an apparent incongruence of data [40]. Previously, reduced *Shh* signaling had been argued to favour the “axis shift” hypothesis. In this context, it was suggested that the presence of a posterior necrotic zone in the early wing could explain the loss of posterior digits in evolution [24]. However, our experimental frameshifts actually suggest the alternative that more posterior morphologies failed to develop, as the result of posterior cells becoming re-specified to more anterior identities. In fact, a large anterior necrotic zone is present in both fore and hind limb buds of mouse and chicken that is unrelated to any evolutionary loss of anterior digits [41]. Mechanistic plausibility is an important pre-requisite for acceptance of any hypothesis, which is now confirmed for the homeotic frameshift. In contrast, the possible mechanisms underlying an “axis shift” remain unclear, and it is yet to be proven experimentally possible. Experimental re-creation of evolutionary events (“synthetic experimental evolution” [42]) is an important new component of evolutionary biology. Because evolution and developmental experiments emerge as twin outputs of the same underlying mechanisms, they often illuminate each other in concrete ways [43]. Our experiments support continued inquiry into regulatory mechanisms that relate Shh signalling and HoxD expression in the limb [44,45]. It is conceivable that specific mutations responsible for inducing the homeotic frameshift may be identified in birds.

## Methods

### Gene cloning and whole-mount in situ hybridization

To make in situ probes, specific gene segments were cloned using exact primers (designed from http://www.ensembl.org/index.html) for Chicken, Mouse and Rabbit. The chosen segments of *HoxD11* and *HoxD12* for rabbit and mouse were identical in sequence (100% homology). The following probes were used:

### Chicken (*Gallus gallus*)

#### HoxD10

TCCCTTCCTACCAGAGGCTGGTGTCTGAATCATGCCCCATNGAGAACCCCGAGGTTCCCGTCCCAGGATATTTTAGACTGAGCCAGACCTACGCCACTGGGAAAACCCAAGAGTACAATAATAGCCCTGAAACGAGTTCAACCGTAATGTTACAGTTAAACCCTCGCGGCAGCTCCAAACCGCAGCTATCTTCTCAACTTCA.

#### HoxD11

TTGCTCTTCTCTGCAACAGCCTCACCGGGAGGGGAAGCGGCGCCGCTCGCCGTTGTCACTTTCTTCTCCTGGCCGGAAGGTGCCTTGCTGCAGGCGCTGTGCTGGGGCTTCAGCTCGCCCTTGTCCGCGTCCCCCTCGCCCTCGGAGTGCTGCTGGTACGGGGGCGCGGGGGCCGGCTCGTAGAACTGGTCGAAGCCCTGCGGCAGGATGCCGTTCCTCCCCACCGAGCCGTAGAAGTTGGAGGCGGCGGGGGACGCTCCGTGGTGGCCGCACACGGGGTCCGTTTTGAATAGCATTTCCGTCCTCCTGTTCGCGGGCTGCAGAAAGTCTCTGTGCATCACCTCTTCAGCTGAGTAATAGGGAGCGTAACTGCCCCTGTACTGCCATTTGCCGCGCTCTAATCCGTATTCCCTGAATGCCACT.

### Rabbit (*Oryctolagus cuniculus*) and mouse (*Mus musculus*)

#### HoxD10

ACAGTTGGACAGACCCGAACAGATCTTGTCGAATAGAGCAACCTGTTACACAGCAAGTCCCCACTTGCTCCTTCACCACCAACATTAAAGAAGAATCCAATTGCTGCATGTATTCTGATAAGCGCAACAAACTCATTTCTGCCGAGGTCCCTTCGTACCAGAGGCTGGTCCCCGAGTCCTGTCCCGTTGAGAACCCTGAGGTTCCTGTCCCTGGATATTTTAGACTGAGTCAGACCTACGCCACCGGGAAAACCCAAGAGTACAATAACAGCCCCGAAGGCAGCTCCACTGTCATGCTCCAGCTCAACCCTCGTGGCGCGGCCAAGCCGCAGCTCTCCGCCGCCCAGCTGCAGATGGAAAAGAAGATGAACGAGCCCGCGAGCGGCCAGGAGCCCACTAAAGTCTCCCA.

#### HoxD12

CTTCGGCGGGCTTGCTCTGCAGTCCTACCTGGCCGGCTCCGGGCCTCTGGGCCTGCAGCCCCCGGGCGCCAAGGACGGACCCGAAGAGCAGGCCAAGTTCTATTCGCCGGAAGCAGCCGCCAGTCCGGAGGAGCGCGGCCGTACGAGGCCGCCCTTCGTCCCAGAGTCTAGCTTGGCCCCTGCAGCCGCTGCTCTCAAGGCCAAATACGACTACGCGGGTATGGGCCGTGCCGCGCCGGGCTCTGCGACCCTGCTCCAGGGGGCCCCCTGCGCCGCCGGCTTCAAGGAGGATACGAAGGGCACGCTCAACTTGAACATGACAGTGCAGGC.

Embryos were collected at day 7 of incubation and fixed during 2 hr to O/N with 4% paraformaldehyde (PFA). Rabbit embryos were collected at 14 dpf, a stage that corresponds with mouse at 13 dpf. Embryos were dehydrated in a methanol series and stored at -20°C. Rehydrated chicken embryos were treated with 6% peroxide solution into PBT by 30 minutes. Mouse and rabbit were rehydrated and treated with acetylation solution (triethanolamine, acetic anhydride and chloridric acid) for 10 and 40 minutes, respectively. Whole mount in situ hybridization was carried out [46].

### Fate-mapping of wing buds

Broiler chicken eggs were incubated at 38°C for 3–3, 5 days and stages were selected [47]. Limb buds cells were labeled with DiI (1, 1-dioctadecyl-3, 3, 3′, 3′-tetramethylindocarbocyanine perchlorate; Sigma-Aldrich) and DiO (3, 3′-dioctadecyloxacarbocyanine, perchlorate; Sigma-Aldrich), fluorescent lipophilic dyes that label the cell membrane and do not leak into neighbouring cells. Both dyes were prepared [48] (DiI 1% in 100% Ethanol, DiO 1% in dimethylformamide) and administered [34,49] by pressure-injection using a Picospritzer®III (Parker Hannifin Corporation; General Valve) and a pulled micropipette with an open tip made with a 0.78 mm (inner diameter) borosilicate capillary. For labelling wing digits, the embryos were selected and injected *in ovo* at stage HH18-19 into the sub-apical region [34]. Immediately after injection the limb bud was photographed in light microscope (Olympus SZX10), cyclopamine was applied, and the embryo put back to incubate at 38°C until HH31, in which the injected wing was photographed under a fluorescence microscope (Olympus BX61) and again under light microscope. This procedure was delivered to both control and cyclopamine-treated embryos. In all these experiments, the cells were labelled in the right limb bud and measurements were taken of the dye dot size and position at the time of administration and after the incubation period. The size range of the injected dye dots was 60–100 μm. Position of injected dots within the limb bud was determined in relation to the position of neighbouring somites [24,34].

### Cyclopamine treatment

Cyclopamine was applied as in previous studies [25,27,28]. Eggs were incubated at 38°C and windowed at day 3–3.5 to obtain embryos spanning stages 18–19 according to Hamburger and Hamilton [47]. We delivered 5 μl of 1 mg/ml solution of Cyclopamine (LC Laboratories) in 45% 2-hydropropyl-b-cyclodextrin (HBC; Sigma) into the amniotic cavity, in direct contact with the embryo [25,27,28]. Presumably, previous procedure of injecting DiO or DiI could have somewhat delayed cyclopamine application, with most applications being delivered towards stage 19.

### Whole-Mount immunofluoerescence

Embryos were fixed in Dent’s fixation solution (4:1 methanol:DMSO) for two hours at room temperature, dehydrated in methanol and left overnight at −80°C to postfix. Large embryos were skinned before immersion in the fixative. Embryos were bleached for 24 h at room temperature in Dent’s bleaching (4:1:1 methanol:DMSO:H2O2). Primary antibody was diluted in PBST, 5% normal goat serum (NGS) and 5% DMSO. The Antibody used was anti collagen type 9 (1:20, DSHB). Immunolabeling was carried out for 48 h at 4°C in agitation. Primary antibodies were washed six times (1 hour each) in PBST and incubated overnight at 4°C with Alexa Fluor 488 goat anti-mouse IgG (H + L) (A-11001, Molecular Probes) or Alexa Fluor 596 goat anti-mouse IgG (H + L) (A-11031, Molecular Probes) as secondary antibodies. The secondary antibodies were washed another six times (1 hour each) in PBST and cleared in Scale [50] for at least five days. Embryos were photographed in stereoscopic fluorescent microscope Olympus MVX10 with a Qimaging camera.

## Competing interests

The authors declare no competing financial interests.

## Authors’ contributions

MSS performed the experiments. CGB assisted MSS in gene cloning and in-situ hybridization. LOF, JFB, and MR contributed with whole-mount immunostaining. AOV designed the experiments and wrote the paper. All authors read and approved the final manuscript.
